# A hyperactive *sleeping beauty *transposase enhances transgenesis in zebrafish embryos

**DOI:** 10.1186/1756-0500-3-282

**Published:** 2010-11-04

**Authors:** Morgan Newman, Michael Lardelli

**Affiliations:** 1 Discipline of Genetics, The School of Molecular and Biomedical Science, The University of Adelaide, Adelaide, SA, 5005, Australia

## Abstract

**Background:**

Transposons are useful molecular tools for transgenesis. The 'sleeping beauty' transposon is a synthetic member of the Tc1/mariner transposon family. Davidson *et al*. (2003) previously described a vector for zebrafish transgenesis consisting of the inverted repeats of 'sleeping beauty' flanking the gene to be transposed. Subsequently, there have been attempts to enhance the transpositional activity of 'sleeping beauty' by increasing the activity of its transposase. Recently, Mates *et al*. (2009) generated a hyperactive transposase giving a 100-fold increased transposition rate in mouse embryos.

**Findings:**

The aim of this experiment was to determine whether this novel hyperactive transposase enhances transgenesis in zebrafish embryos. Using our previously characterised *mitfa-*amyloidβ-GFP transgene, we observed an eight-fold enhancement in transient transgenesis following detection of transgene expression in melanophores by whole mount *in-situ *hybridisation. However, high rates of defective embryogenesis were also observed.

**Conclusion:**

The novel hyperactive 'sleeping beauty' transposase enhances the rate of transgenesis in zebrafish embryos.

## Findings

Transposons direct integrations of single copies of genetic material into chromosomes [1] and are useful molecular tools for transgenesis in vertebrate species. They function by delivering a gene of interest to the chromosome in a cut and paste manner. The 'sleeping beauty' transposon is a synthetic member of the Tc1/mariner transposon family. The transposon was engineered from a consensus sequence of inactive fossil transposon sequences from various Salmonid fish genomes [2]. Sleeping beauty consists of the transposase gene flanked by terminal inverted repeats of direct repeats. The transposase protein catalyses the excision and integration of donor DNA into a TA dinucleotide site of a recipient genome [1]. The derived sleeping beauty vector system (SBT) has been shown to enhance production of transgeneic animals in comparison to simple methods of transgenesis such as injection of naked DNA [3,4]. It is active in various vertebrate species such as fish, frogs, mice and rats [3,5-7]. There have been attempts to enhance the transpositional activity of the SBT, specifically by increasing the activity of the transposase. Almost every amino acid has been changed to derive hyperactive mutants of the SB transposase and this has yielded modest increases in transpositional activity [8-11]. Recently, Mates et al. [5], used a large-scale genetic screen in mammalian cells to generate a hyperactive transposase that gave a ~100-fold enhancement of transpositional activity over the original SB transposase in mouse embryos.

Alzheimer's disease may be caused by the accumulation of amyloidβ peptides in the brain [12]. Recently, we used the SBT system to generate a zebrafish melanophore model of amyloidβ toxicity [7]. We generated transgenic zebrafish possessing human amyloidβ under the control of the *mitfa *promoter that drives expression specifically in melanophores (dark pigment cells) using our vector pT2-*mitfa-*amyloidβ-GFP. In that study the transposase mRNA was generated from the plasmid pSBRNAX that includes sequence from the 3' UTR of the *Xenopus *β-globin gene for mRNA stabilisation. In this experiment, we compared the rates of transient transgenesis in zebrafish embryos using the original transposase mRNA (SB10, generated from pSBRNAX [3]) or the hyperactive transposase mRNA (SB100, generated from pCMV(CAT)T7-SB100X [5]). It is important to note that the pCMV(CAT)T7-SB100X vector does not contain the *Xenopus *β-globin 3' UTR sequences for mRNA stabilisation. Therefore, SB100 mRNA may not be as stable as SB10 mRNA and, once injected into the zebrafish embryos, may possibly degrade at a faster rate.

Zebrafish embryos were injected at the 1-cell stage with ~3 nl of linearised pT2-*mitfa-*amyloidβ-GFP mixed with either SB100 or SB10 transposase mRNA (final concentration of DNA and mRNA is 25 ng/μl each) [6]. Embryos were permitted to develop until ~24 hour post-fertilisation (hpf) at which time their chorions were removed and they were fixed in 4% formaldehyde in a phosphate buffered saline solution. There was some variability in the normal development of individual embryos injected with the SB100 mRNA. From a total of 58 injected embryos, 22 showed developmental defects. Specifically, 6 showed defects in epiboly (but continued to develop later stage tissues) and 16 had trunk/somitogenesis development defects (Table 1).

**Table 1:** Results of injections of transposase mRNAs alone or with the *mitfa*-amyloidβ-GFP transgene

Injection	Normal % (n)	Mild % (n)	Severe % (n)
Uninjected	98 (59)	2 (1)	0
*mitfa-*amyloidβ-GFP only	94 (60)	3 (2)	3 (2)
-with SB100 mRNA	62 (36)	28 (16)	10 (6)
-with SB10 mRNA	97 (59)	3 (2)	0
SB100 mRNA only	40 (20)	16 (8)	44 (22)
SB10 mRNA only	83 (39)	8.5 (4)	8.5 (4)
TOL2 mRNA only	83 (38)	8.5 (4)	8.5 (4)

Percentage of embryos at 24 hpf with the number of embryos observed in parentheses.

Normal: wild type appearance. Mild: trunk/somitogenesis-like defects. Severe: epiboly-like defects.

**Figure 1:**
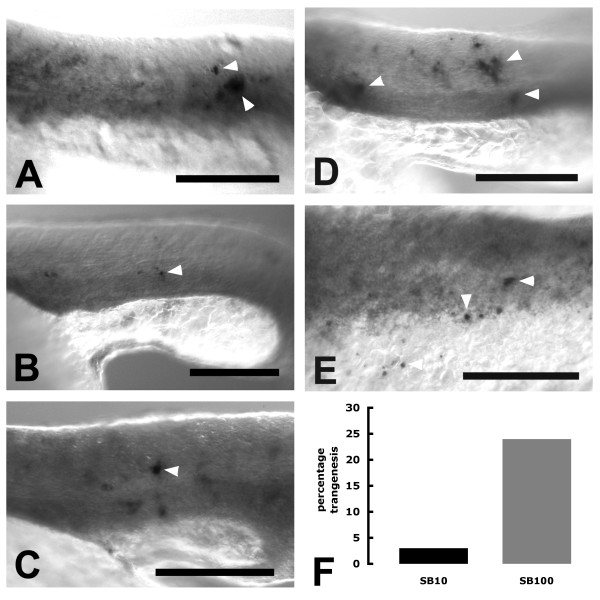
**Transgene expression after use of SB100x transposase**. Embryos injected with transgene pT2-*mitfa-*amyloidβ-GFP and SB100x mRNA were examined at 24 hpf for transgene expression by whole mount *in situ *transcript hybridisation against GFP sequences included in the transcript. A-D, lateral views of trunk region of embryos. E, dorsolateral view of hindbrain/spinal cord region of embryo. White arrowheads indicate some of the cells apparently expressing the transgene in a pattern consistent with expression in future melanophores in which the *mitfa *promoter is active. Size bars indicate approximately 100 uM. Deformities caused by the SB100 transposase mean that some structures (e.g. the yolk extension) are an inconsistent size. F, Histogram showing percentage rates of transient transgenesis driven by SB10 and SB100 transposases.

Whole-mount *in-situ *transcript hybridization (WISH) was then performed on fixed embryos essentially as described by Jowett [13]. Since the GFP coding sequences in the pT2-*mitfa-*amyloidβ-GFP transgene are transcribed and not translated a digoxigenin-labelled antisense EGFP cRNA probe was used, as previously described [7], to detect cells transcribing GFP (in general, this can also provide more sensitive detection of gene expression than observation of GFP fluorescence). Fixed embryos were stained for ~18 hours at 4°C, followed by ~6 hours at room temperature to be confident that all putative melanophores expressing the GFP transcript were revealed. There was some variability in the number of putative melanophores expressing the GFP transcript in individual embryos. However, of the 58 SB100 mRNA injected embryos, 14 (24%) (Figure 1F) had putative melanophores expressing the GFP transcript (see figure 1A-E) and of the 61 SB10 mRNA injected embryos, only 2 (3%) (Figure 1F) had putative melanophores expressing the GFP transcript. Therefore, injection of the SB100 mRNA resulted in an 8-fold enhancement of transient transgenesis in zebrafish embryos. Interestingly, 10 out of 14, SB100 mRNA injected embryos with GFP transcript expression, also had the above mentioned trunk/somitogenesis development defects. This is consistent with higher rates of transgenesis being associated with higher rates of deformity [14]. To determine whether injection of the SB100 mRNA by itself might cause developmental defects, we determined the relative rates of defective embryos from injections of only linearised pT2-*mitfa-*amyloidβ-GFP DNA, SB10 mRNA, SB100 mRNA or another transposase mRNA transcribed from the pCS-TP plasmid [15] (TOL2 mRNA) at 25 ng/μl. The results in Table 1 clearly show that only the SB100 mRNA causes increased developmental defects, indicating that the SB100 mRNA and not the transgene causes this. Embryos with epiboly defects are arrested in development at a stage before differentiation of melanophores is expected. Therefore, it is not possible to observe melanophore-specific GFP expression in these embryos. However, the possibility exists that these embryos also possess the transgene. Testing of the effects of a range of SB100 mRNA injection concentrations will be necessary to determine which concentration gives the optimum balance between transgenesis and embryo defect rates.

In their tests of SB100-driven transgenesis in fertilised mouse oocytes, Mates *et al*. (2009) did not observe a decreased survival rate relative to uninjected controls at day 7 of mouse embryogenesis and high rates of transgenesis were observed in mouse litters. However, the slower rate of cell division that occurs in cleavage stage mouse embryos relative to zebrafish embryos may mean that the transposase mRNA breaks down in the mouse zygotes before it can cause developmental defects.

The enhancement of transgenesis in zebrafish embryos from use of the novel hyperactive transposase was not ~100-fold greater than the transgenesis rate using the original SB transposase. Nevertheless, the observed 8-fold increase is a considerable improvement for two reasons. First, the amyloidβ-GFP transgene is under the control of a tissue-specific promoter, *mitfa*, which directs expression of the transgene to melanophores. Melanophores make up only a small fraction of the total cells in a zebrafish embryo at 24 hpf. Thus, transient transgenesis is not expected to label this cell type frequently. Secondly, the SB10 mRNA is generated from pSBRNAX which has the *Xenopus *β-globin 3' UTR sequence for increased mRNA stability while the SB100 mRNA does not include such sequences. Therefore, the SB100 mRNA would be expected to degrade at a faster rate which might also affect transgenesis efficiency. If one considers that the germline transmission frequency of *mitfa-*amyloidβ-GFP in the original study using SB10 mRNA was 20% (for a <3% rate of observable transient transgenesis), then the 8-fold enhancement of transient transgenesis observed in this study would presumably further improve the rate of germline transgenesis in zebrafish. Overall, we conclude that the novel hyperactive 'sleeping beauty' transposase enhances the rate of transgenesis in zebrafish embryos.

## Competing interests

The authors declare that they have no competing interests.

## Authors' contributions

MN completed experiments, participated in the design of the study and data analysis and drafted the manuscript. ML predominantly designed the study, participated in data analysis and revisions of the manuscript.
